# Biological functions and clinical applications of exosomal long non‐coding RNAs in cancer

**DOI:** 10.1111/jcmm.15873

**Published:** 2020-09-14

**Authors:** Yali Wang, Mengdi Zhang, Fangfang Zhou

**Affiliations:** ^1^ MOE Laboratory of Biosystems Homeostasis & Protection and Innovation Center for Cell Signaling Network Life Sciences Institute Zhejiang University Hangzhou China; ^2^ Institutes of Biology and Medical Science Soochow University Suzhou China

**Keywords:** cancer, exosomal lncRNA, exosome

## Abstract

Exosomes are extracellular vesicles secreted by donor cells, and one of the important roles of exosomes is intercellular communication. Exosomes contain proteins, lipids, DNA and RNA. The components exert their functions by modulating the cellular processes of recipient cells. Exosomal long non‐coding RNAs (lncRNAs) are important components and play multiple roles in tumorigenesis and tumour development. In this review, we summarize the biological functions and clinical applications of exosomal lncRNAs in cancer. Exosomal lncRNAs regulate cell proliferation, metastasis, drug resistance and angiogenesis in human cancers. Since exosomal lncRNAs are associated with clinicopathological characteristics of cancer, these might be potentially useful biomarkers for diagnosis and prognosis of cancer. Exosomal lncRNAs participate in multiple processes of cancer progression, which makes them promising therapeutic targets for cancer treatment.

## 1 INTRODUCTION

Exosomes are microvesicles that are derived from multivesicular bodies (MVBs) and released into the extracellular space upon fusion of MVBs with the plasma membrane.^1^ Exosomes contain multiple components including lipids, proteins, RNA and DNA. Exosomes take part in the intercellular communication by transferring cargoes from donor cells to recipient cells.

One of the cargoes of exosomes is long non‐coding RNA (lncRNA). LncRNAs are RNA transcripts longer than 200 nt and have limited protein‐coding potential.^2^ LncRNAs are involved in numerous cellular processes. LncRNAs participate in the pathogenesis of many diseases, including cancer.^3^ Lots of studies have demonstrated that lncRNAs regulate the malignant characteristics of cancer such as metastasis and drug resistance. Exosomal lncRNAs are RNA molecules, and exosomal lncRNAs acquired by recipient cells will exert their cancer‐related roles in the recipient cells to regulate cancer progression. In this review, we summarize recent research regarding exosomal lncRNAs in cancers. We describe the biological roles of exosomal lncRNAs in cancer and discuss the potential clinical applications of exosomal lncRNAs in the future.

## 2 EXOSOMES

Exosomes are extracellular vesicles with a diameter of 30‐100 nm and are released by multiple types of cells.^4^, ^5^, ^6^ In the 1980s, exosomes were observed during reticulocyte maturation.^7^, ^8^ The production of exosomes begins with a process called endocytosis.^9^

**Figure 1:**
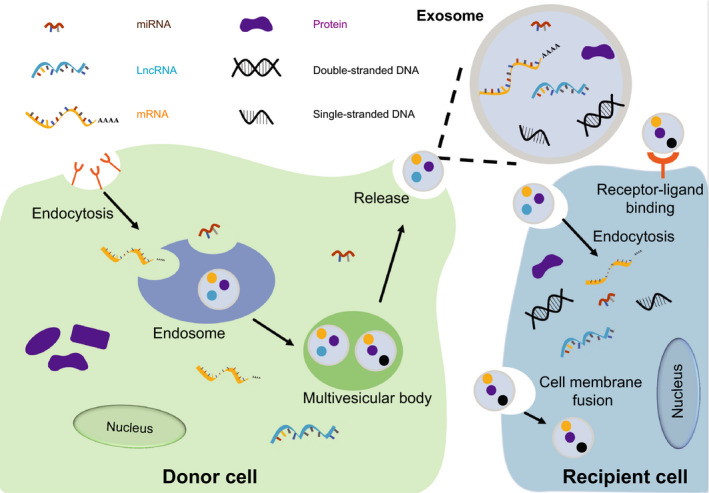
The intercellular communication performed by exosomes. The inward budding of cell membrane results in the formation of endosome. The further inward budding of endosome membrane results in multivesicular body (MVB) formation, then MVBs fuse with cell membrane and release exosomes to extracellular space. The exosomes are received by recipient cells, and the cargoes (DNAs, RNAs, proteins) contained in exosome exert function in recipient cells

Exosomes are derived from inward budding of the plasma membrane. The inward budding of the plasma membrane forms an endosome. Further inward budding of the membrane results in the formation of intraluminal vesicles (ILVs) inside the MVB. Then, the MVB fuses with the plasma membrane and releases the ILVs called exosomes to the extracellular milieu (Figure 1).

Various factors take part in the formation of exosomes, such as proteins and lncRNAs.^10^, ^11^

Rab GTPases regulate the biogenesis and secretion of exosomes.^12^ Rab5b plays a role in the motility and fusion of early endosomes.^13^ Rab35 regulates MVB transport and controls the docking process. Rab35 depletion increases intracellular accumulation of endosomal vesicles and decreases exosome secretion.^14^ Soluble N‐ethylmaleimide‐sensitive factor attachment protein receptors (SNAREs) are trans‐membrane proteins and SNARE complexes mediate membrane fusion and regulate the release of exosomes. Ternary SNARE complexes consist of a SNARE on vesicle membrane (v‐SNARE) and two SNAREs on target membrane (t‐SNARE).^15^, ^16^ Synaptosomal‐associated protein (SNAP) such as SNAP23 is t‐SNAREs and vesicle‐associated membrane protein (VAMP) such as VAMP3 and VAMP8 are v‐SNAREs.^17^, ^18^, ^19^ The phosphorylation of SNAP23 enhanced the stability of the SNARE complex and promoted the secretion of exosomes.^20^, ^21^

LncRNA‐APC1 regulates the production of exosomes by interacting with Rab5b mRNA.^22^ The interplay of lncRNA‐APC and Rab5b mRNA reduces the stability of Rab5b mRNA and inhibits Rab5b expression, leading to a reduction in exosomes. On the contrary, HOTAIR enhances the release of exosomes by modulation of several processes.^23^ It regulates the docking process by modulating Rab35 expression and localization. In addition, HOTAIR facilitates the fusion process by controlling the colocalization of VAMP3 and SNAP23. HOTAIR also enhances the release of exosomes via phosphorylation of SNAP23.

Exosomes contain multiple bioactive molecules, including lipids, proteins, RNA and DNA.^24^, ^25^, ^26^, ^27^ The components of plasma membranes such as cholesterol, sphingomyelin, hexosylceramides, phosphatidylserine and saturated fatty acids are also present in the exosomes.^28^ Rab GTPases and annexins, the proteins associated with membrane transport and fusion, are found abundantly in the exosomes. ESCRT components, ALIX and TSG101 are consistently detected in exosomes. Moreover, exosomes are enriched in heat‐shock proteins, HSP70 and HSP90; tetraspanins, including CD9, CD63, CD81 and CD82; MHC class II proteins; members of the human epidermal receptor family; and epithelial cell adhesion molecules.^29^, ^30^, ^31^, ^32^, ^33^ LncRNAs lncARSR and LNMAT2 have been reported in the exosomes derived from cancer cells.^34^, ^35^ Various investigations have revealed the presence of miRNAs, such as miR‐21 and miR‐221 in the exosomes.^36^, ^37^ Single‐stranded DNA and double‐stranded DNA are also found in the exosomes.^38^, ^39^

Earlier, exosomes were regarded as cellular garbage bags with non‐functional cellular molecules or excess constituents. Emerging studies demonstrated the various functional roles of exosomes in physiological and pathological processes.^40^, ^41^

As exosomes released from donor cells are accepted by recipient cells, one of the most important roles of exosomes is intercellular communication. Exosomes contain multiple biological active molecules and the message included in cargoes was transferred from one cell to another. Exosomal components, such as proteins, miRNAs and lncRNAs regulate the biological processes of recipient cells.

The cargoes inside the exosomes reflect the pathophysiological state of the donor cells. Exosomal constitution may vary depending on the donor cells.^25^, ^27^, ^42^, ^43^ Moreover, exosomes are detected in multiple physiological fluids, including plasma or serum, saliva, amniotic fluid, breast milk, urine, nasal secretion, cerebrospinal fluid, semen and pathological fluids such as ascitic fluid.^44^ The wide existence of exosomes makes it convenient to employ the exosomes into the non‐invasive diagnosis of diseases, including human cancers.^27^, ^45^, ^46^, ^47^, ^48^

## 3 LNCRNAS

**Figure 2:**
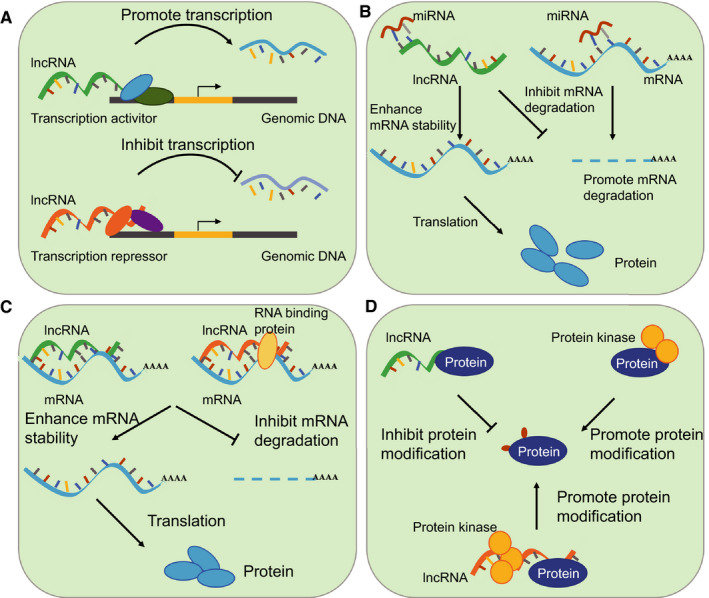
Regulatory roles of lncRNAs. A, LncRNAs recruit transcript factors or epigenetic regulators to gene promoter region and regulate gene transcription. B, LncRNAs competitively bind to miRNAs and inhibit the degradation of miRNAs target genes. C, LncRNAs interact with mRNA directly or enhance the interplay of mRNA and protein, which promotes the mRNA stability and increases gene expression. D, LncRNAs interact with protein and inhibit protein modification. LncRNAs interact with protein kinase and enhance protein modification

LncRNAs are a type of transcripts with a length >200nt and limited protein‐coding potential. LncRNAs are reported to take part in multiple cellular processes through various mechanisms. LncRNAs interact with miRNA, mRNA, protein and genomic DNA, and subsequently regulate gene transcription, translation, mRNA stability, protein modification and the interplay of protein with other factors^49^, ^50^, ^51^, ^52^, ^53^, ^54^, ^55^, ^56^ (Figure 2). LncRNAs inhibit the degradation of miRNA target genes by interacting with miRNAs. The expression of ZEB1, a target gene of miR‐200 was found to be enhanced through the interaction of LncRNA‐ATB with miR‐200.^52^ LncRNAs recruit transcription factors or epigenetic modulators to the chromatin region or genomic DNA to regulate gene expression. In cancer cells, the oncogene EPIC1 interacts with MYC and regulates the occupancy of MYC on target promoters.^57^

LncRNAs also play important roles in disease occurrence and tumour progression. They are found to regulate cell proliferation, migration and invasion of cancer cells, and modulate cell cycle, apoptosis, lymphatic metastasis and drug sensitivity.^58^, ^59^, ^60^, ^61^ VEGF‐C is a critical factor in lymphatic metastasis of human cancers.^62^, ^63^ It is overexpressed in many types of cancers and associated with regional lymph node metastasis and poor survival. LncRNA BLACAT2 has been reported to promote lymphatic metastasis of bladder cancer via regulating VEGF‐C expression.^64^ WDR5 is a core subunit of H3K4 methyltransferase complexes and a binding protein of BLACAT2. BLACAT2 interacts with WDR5 and enhances the H3K4me3 levels of VEGF‐C promoter thereby up‐regulating VEGF‐C expression. Another lncRNA in bladder cancer, LNMAT1, promotes lymphatic metastasis through modulation of tumour microenvironment.^65^ LNMAT1 overexpression is seen in bladder cancer with lymph node metastasis and associated with poor survival. In addition, LNMAT1 can recruit hnRNPL to CCL2 promoter and up‐regulate the levels of H3K4me3 of this region, thereby increasing the expression of CCL2. Increased CCL2 recruits macrophages to the tumour which promotes lymphatic metastasis via VEGF‐C secretion. Furthermore, the up‐regulation of LncRNA GMAN is associated with metastasis in gastric cancer. Thus, high levels of GMAN predict poor survival in patients. Previous studies have found that GMAN enhances the translation of Ephrin A1 via interaction with GMAN‐AS.^66^

## 4 EXOSOMAL LNCRNAS IN CANCER—BIOLOGICAL FUNCTIONS

LncRNAs play important roles in cancer progression through multiple mechanisms. Exosomal lncRNAs are transferred to recipient cells and exert oncogenic roles or tumour suppressive roles in the recipient cells (Table 1).^67^, ^68^, ^69^, ^70^, ^71^

**Table 1:** The function and application of exosomal lncRNAs in cancer

LncRNA	Type of cancer	Donor cell/recipient cell	Biological function	Clinical application	Reference
lncARSR	Renal cancer	Drug‐resistant cells/drug‐sensitive cells	Promote sunitinib resistance	Therapeutic target	^24^
LNMAT2	Bladder cancer	Cancer cells/HLECs	Promote lymphatic metastasis	Therapeutic target	^25^
MALAT1	Colorectal cancer	Metastatic cancer cells/primary cancer cells	Promote metastasis	Therapeutic target	^67^
RPPH1	Colorectal cancer	Cancer cells/macrophage M2	Promote metastasis	Therapeutic and diagnostic target	^68^
GAS5	Lung Cancer	Cancer cells/HUVECs	Promotes angiogenesis	Therapeutic target	^82^
AFAP1‐AS1	Breast cancer	Drug‐resistant cells/drug‐sensitive cells	Promote trastuzumab resistance	Therapeutic target	^75^
SNHG14	Breast cancer	Drug‐resistant cells/drug‐sensitive cells	Promote trastuzumab resistance	Therapeutic target	^76^
LINC00461	Multiple myeloma cell	Mesenchymal stromal cells/multiple myeloma cells	Promote proliferation and suppress apoptosis	Therapeutic target	^63^
PART1	ESCC	Drug‐resistant cells/drug‐sensitive cells	Promote gefitinib resistance	Therapeutic target	^73^
PCAT1	ESCC	Cancer cells/immortalized normal oesophageal epithelial cells	Promote proliferation	Biomarker	^64^
TIRY	Oral squamous cell carcinoma	Cancer‐associated fibroblast/cancer cells	Promote metastasis	Therapeutic target	^70^
SOX2OT	Pancreatic ductal adenocarcinoma	Cancer cells/cancer cells	Promote metastasis	Biomarker for prognosis	^66^
UCF1	NSCLC	Cancer cells/cancer cells	Promote proliferation, migration and invasion	Biomarker for diagnosis	^60^
UCA1	NSCLC	Drug‐resistant cells/drug‐sensitive cells	Promote gefitinib resistance	Therapeutic target	^71^
H19	NSCLC	Drug‐resistant cells/drug‐sensitive cells	Promote gefitinib resistance	Therapeutic target	^72^
HOTTIP	Gastric cancer	Drug‐resistant cells/drug‐sensitive cells	Promote cisplatin resistance	Therapeutic target	^78^
TUG1	Cervical cancer	Cancer cells/HUVECs	Promote angiogenesis	Target for early diagnosis	^81^
CCAT2	Glioma	Cancer cells/HUVECs	Promote angiogenesis	Therapeutic target	^83^
LINC‐POU3F3	Glioma	Cancer cells/human brain microvascular endothelial cells	Promote angiogenesis	Therapeutic target	^84^

### 4.1 Proliferation

Excessive growth of cancer cells is a fundamental characteristic of human cancers. Cancer‐associated genes typically participate in cell proliferation to regulate cell growth. Exosomal lncRNAs are involved in this process.

LncRNA UFC1 is up‐regulated in non‐small cell lung cancer (NSCLC) tissues and serum exosomes of NSCLC patients. It has been found that UFC1 promotes the proliferation of NSCLC cells through modulating the expression of PTEN. It interacts with EZH2 to regulate the H3K27me3 levels of PTEN promoter region. Exosomal UFC1 is transferred to receptor cells and induces their growth.^72^

MALAT1 from exosomes promotes the proliferation of breast cancer cells and NSCLC cells.^73^, ^74^ In multiple myeloma, exosomal LINC00461 has been reported to enhance cell proliferation via binding to miR‐15a/16 and promoting the expression of BCL‐2.^75^ Similarly, in oesophageal squamous cell carcinoma (ESCC) cells, exosomal lncRNA PCAT1 promotes cell proliferation through binding to miR‐326.^76^ Exosomal ZFAS1 also promotes the cell proliferation of ESCC cells by regulating the miR‐124/STAT3 axis.^77^

### 4.2 Metastasis

Metastasis is one of the major malignant characteristics of cancer, and patients with metastatic cancer have a poor prognosis. Lymphangiogenesis and epithelial‐mesenchymal transition (EMT) facilitate the process of cancer metastasis.

LncRNA SOX2OT is reported to take part in the progress of several cancers, including lung cancer, breast cancer, ESCC and pancreatic ductal adenocarcinoma (PDAC). In PDAC cells, SOX2OT promotes SOX2 expression by binding to miR‐200 and facilitates EMT and stem cell like properties. The exosomal SOX2OT from PDAC cells enhances the metastasis of acquired PDAC cells.^78^

MALAT1 is overexpressed in several cancer cells and regulates tumour development. In a previous study, exosomal MALAT1 derived from metastatic colorectal cancer (CRC) cells was found to promote the expression of FUT4 in the primary CRC cells through binding to miR‐26a and miR‐26b.^79^

In CRC, lncRNA RPPH1 interacts with TUBB3 to inhibit its ubiquitination and promotes EMT of CRC cells.^80^ In addition to regulating metastasis, the exosomal RPPH1 from CRC cells also regulates the behaviour of macrophages. Exosomal RPPH1 mediates macrophage M2 polarization to induce metastasis in CRC.

Lymph node metastasis is associated with poor survival in patients with bladder cancer. It has been found that in bladder cancer, lncRNA LNMAT2 is incorporated into exosomes through binding with hnRNPA2B1.^35^ A previous report demonstrated that the exosomes derived from bladder cancer cells were transmitted to human lymphatic endothelial cells (HLECs). Subsequently, LNMAT2 increased the expression of PROX1 via epigenetic modulation of H3K4me3 levels in PROX1 promoter sequences. Thus, it was suggested that exosomal LNMAT2 promoted lymphangiogenesis and lymph node metastasis in bladder cancers.

Thyroid cancer is one of the most lethal human cancers. Thyroid cancer stem‐like cells (CSCs) are significantly associated with tumorigenesis. LINC‐ROR plays multiple roles in several kinds of cancers, such as regulating the proliferation and drug sensitivity of hepatocellular carcinoma, promoting metastasis of pancreatic cancer and enhancing the EMT in ovarian cancer cells. In thyroid cancer, CSCs‐derived exosomes containing LINC‐ROR have been found to promote the EMT when transferred to normal cells.^81^

Not only the exosomes from cancer cells but also exosomes from other kinds of cells take part in cancer metastasis. Exosomes from cancer‐associated fibroblasts contain up‐regulated lncRNA TIRY, which regulates the Wnt/β‐catenin signalling pathway by binding to miR‐14 and enhances metastasis in oral squamous cell carcinoma.^82^

In summary, exosomal lncRNAs regulate the metastasis of cancer cells through the following ways: cancer cell‐derived exosomes are transferred to cancer cells and exosomal lncRNAs regulate the metastatic property of cancer cells; cancer cell‐derived exosomes transferred to non‐cancer cells (normal cells/HLECs/macrophage) enhances metastatic potential via exosomal lncRNAs; non‐cancer cell (cancer‐associated fibroblast)‐derived exosomes transferred to cancer cells regulate their metastasis via lncRNAs.

### 4.3 Chemoresistance

**Figure 3:**
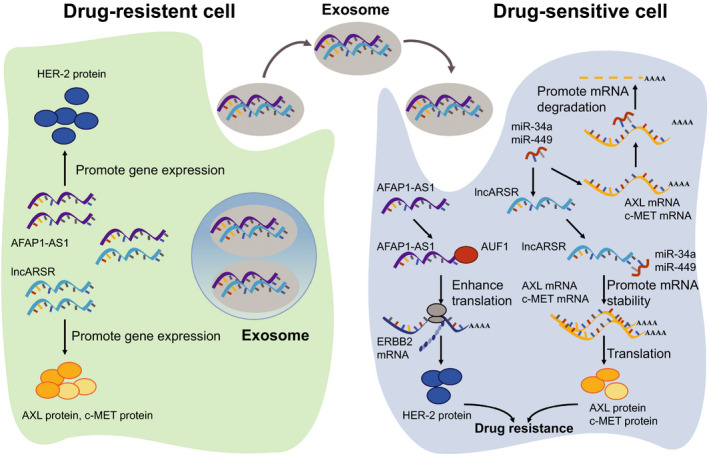
Exosomal lncRNAs transfer chemoresistance in cancer. Exosomal LncRNAs derived from drug‐resistant cells are received by drug‐sensitive cells and promote drug resistance by regulating the expression of drug resistance related genes in sensitive cells. In renal cancer cells, exosomal lncARSR derived from sunitinib resistant cells are transferred to sensitive cells. Exosomal lncARSR competitively bind to miR‐34 and miR‐449 and enhance AXL and c‐MET expression. In breast cancer, exosomal AFAP1‐AS1 transfers the trastuzumab resistance from resistant cells to sensitive cells. Exosomal AFAP1‐AS1 interacts with AUF1 and the interaction promote ERBB2 translation

Drug resistance is a big challenge in cancer treatment and understanding the underlying mechanism of drug resistance will provide therapeutic strategy for cancer patients with drug resistance. Exosomal lncRNAs were reported to take part in drug resistance (Figure 3).

Sunitinib is an oral drug used for treating advanced renal cell carcinoma (RCC). Most of the patients receiving sunitinib therapy develop drug resistance after 6‐15 months of treatment. It is reported that exosomal lncRNAs are involved in the modulation of drug resistance. LncARSR regulates the expression of AXL and c‐MET by binding to miR‐34/miR‐449 and thereby promotes sunitinib resistance in RCC cells.^34^ Exosomal lncARSR from sunitinib‐resistant RCC cells was found to induce drug resistance in previously sensitive cells.

Gefitinib is a type of tyrosine kinase inhibitor that blocks epidermal growth factor receptor tyrosine kinase (EGFR‐TK). In NSCLC, overexpressed lncRNA UCA1 was reported to be responsible for gefitinib resistance.^83^ Moreover, FOSL2 was found to be up‐regulated in gefitinib‐resistant NSCLC cells and UCA1 protected FOSL2 by sponging miR‐143 in NSCLC cells. Some previous studies showed that gefitinib‐resistant NSCLC cells transferred exosomal UCA1 to drug‐sensitive cells which then acquired gefitinib resistance. Along with UCA1, H19 present in the exosomes of NSCLC also modulates gefitinib resistance.^84^ In ESCC, up‐regulated lncRNA PART1 promotes gefitinib resistance by modulating BCL‐2 expression. Exosomes containing PART1 disseminate drug resistance to other sensitive ESCC cells.^85^

Breast cancer is the most common cancer and one of the leading causes of cancer death in females worldwide. Trastuzumab is a humanized monoclonal antibody used in the treatment of HER2‐positive breast cancer patients. Over half of the patients develop drug resistance after trastuzumab therapy. Exosomal AGAP1‐AS1, AFAP1‐AS1 and SNHG14 are reported to participate in the regulation of trastuzumab resistance in HER‐positive breast cancer.^86^, ^87^, ^88^ It is suggested that AFAP1‐AS1 enhances the translation of ERBB2 by associating with AUF1, whereas SNHG14 regulates the BCL‐2/BAX signalling pathway in breast cancer cells.

Cisplatin is an extensively used chemotherapeutic drug for many cancers, such as ovarian cancer, gastric cancer and cervical cancer. In ovarian cancer, exosomal UCA1 promotes cisplatin resistance by modulating the expression of FOSL2.^89^ Exosomal HOTTIP regulates the expression of HMGA1 through binding to miR‐218, thereby promoting cisplatin resistance in gastric cancer.^90^ Additional, HNF1A‐AS1 binds to miR‐34b to up‐regulate TUFT1 expression and subsequently promotes cisplatin resistance in cervical cancer.^91^

In general, lncRNAs serve as ceRNAs to interact with miRNAs and then protect the target genes of miRNAs, that are considered vital in drug resistance. As a result, these lncRNAs are incorporated into the exosomes which then induce drug resistance in previously sensitive cells.

### 4.4 Angiogenesis

Exosomal RAMP‐AS1 is up‐regulated in chondrosarcoma patients and high levels of RAMP‐AS1 are often associated with poor survival. RAMP‐AS1 acts as a ceRNA to bind to miR‐2355‐5p and enhances the expression of VEGFR2, an important factor in angiogenesis.^92^

Cervical cancer cells secrete exosomes containing lncRNA TUG1. Researchers observed that when exosomal TUG1 was transferred to the human umbilical vein endothelial cells (HUVECs), it promoted HUVECs proliferation via inhibiting caspase‐3 activity and regulating apoptosis‐related proteins. Thus, it was suggested that exosomal TUG1 plays a role in angiogenesis.^93^

GAS5 is down‐regulated in lung cancer tissues. The exosomal GAS5 binds to miR‐29‐3p and increases the expression of PTEN. Additionally, GAS5 decreases the phosphorylation of AKT and PI3K. To summarize, exosomal GAS5 represses HUVECs proliferation and enhances their apoptosis, thus, inhibiting angiogenesis in lung cancer.^94^

In glioma, exosomal lncRNA CCAT2 transferred to HUVECs is shown to activate VEGFA and TGFβ. CCAT2 increased BCL‐2 expression while suppressed the expression of BAx and caspase‐3, which inhibited apoptosis. Overall, exosomal CCAT2 promotes angiogenesis.^95^ Another report found that gliomas can induce angiogenesis via secreting exosomes enriched in LINC‐POU3F3.^96^

## 5 EXOSOMAL LNCRNAS IN CANCER—CLINICAL APPLICATIONS

The variations in the composition and expression of exosomal lncRNAs typically reflect the changes in the clinical status of the cell. A wide variety of exosomal lncRNAs take part in tumorigenesis and tumour development, making them potential biomarkers and therapeutic targets for diagnosis, prognosis and therapy of cancer.^97^, ^98^

### 5.1 Diagnostic and prognostic biomarkers

**Figure 4:**
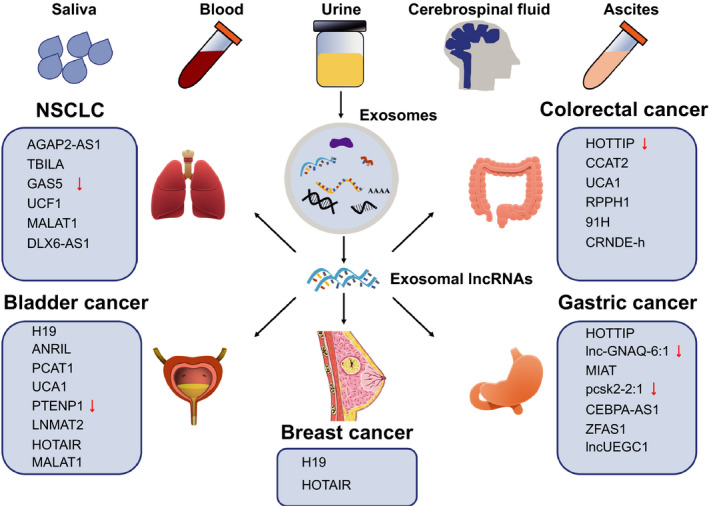
The clinical application of exosomal lncRNAs in cancer. Exosomal lncRNAs from human fluids will serve as biomarkers for diagnosis and prognosis of cancer

Exosomes from serum, urine and other body fluids usually reflect the clinical condition of patients with cancer. The alteration of exosomal lncRNAs may draw a clue to tumour stage. Detailed investigations in exosomal lncRNAs provide more choices for cancer diagnosis and prognosis (Figure 4).

Exosomal H19 is overexpressed in patients with breast cancer compared to healthy individuals and those with benign breast disease. Statistical analysis found that the high expression levels of exosomal H19 are associated with lymph node metastasis, advanced TNM stages and distant metastasis. It has been observed that exosomal H19 decreases in patients with breast cancer post‐surgery. Thus, exosomal H19 can be a potentially useful biomarker for the diagnosis of breast cancer.^99^ Similar observations have been reported for bladder cancer. Higher levels of exosomal H19 have been detected in preoperative samples compared with those in the postoperative samples. Furthermore, levels of exosomal H19 are lower in healthy individuals and patients with benign disease compared with those with bladder cancer. Bladder cancer patients with high level of exosomal H19 generally have poor survival.^100^ In brief, serum exosomal H19 can serve as a non‐invasive diagnostic and prognostic biomarker for patients with breast cancer or bladder cancer.

Exosomal HOTAIR is up‐regulated in patients with laryngeal squamous cell carcinoma (LSCC). Serum exosomal HOTAIR correlates with clinical parameters including lymph node metastasis, clinical stages, and T classification. Exosomal HOTAIR may be a prognostic biomarker for LSCC patients.^101^ In patients with breast cancer, high levels of serum exosomal HOTAIR have been detected as compared to those in healthy individuals. In addition, exosomal HOTAIR is down‐regulated in patients with breast cancer post‐surgery. A high level of exosomal HOTAIR is correlated with poor disease‐free survival and overall survival. Patients with high level of exosomal HOTAIR usually demonstrate a poor response to neoadjuvant chemotherapy and tamoxifen hormonal therapy. Exosomal HOTAIR is further correlated with the expression level of ErbB2. These correlations indicate that exosomal HOTAIR might be a promising liquid biopsy biomarker for breast cancer.^102^

The expression levels of exosomal HOTTIP in gastric cancer patients are higher than those in healthy individuals. Patients with high exosomal HOTTIP have poor overall survival. Considering this, COX analysis found that up‐regulated exosomal HOTTIP can be an independent prognostic factor in patients with gastric cancer.^103^ Contrastingly in CRC, exosomal HOTTIP plays different roles.^104^ Exosomes from CRC patients demonstrate lower expression level of HOTTIP than those from healthy individuals. As opposed to gastric cancer, patients with low level of exosomal HOTTIP have poor overall survival.

The exosomal lncRNAs reflect clinical status in cancer patients. In different types of cancer, specific exosomal lncRNAs reflect different pathophysiological states. Multiple studies are needed to determine the indicative roles of exosomal lncRNA in cancer.

### 5.2 Therapeutic target

Exosomal lncRNAs play important roles in cancer progression. A detailed investigation of the functional roles of exosomal lncRNAs will facilitate their development as promising therapeutic targets for cancer treatment.

LncARSR is an oncogene in RCC and the exosomal lncARSR transfers drug resistance to the drug‐sensitive cells. In vivo study, nude mice were orthotopically xenografted with sunitinib‐resistant RCC cells or subcutaneously xenografted with sunitinib‐resistant patient‐derived xenograft, and the treatment was performed by intravenous injection or intratumoural injection of lncARSR locked nucleic acids (LNAs), respectively. Studies have shown that LNAs targeting lncARSR reduced the expression of lncARSR and restored the sunitinib response. LncARSR served as a promising therapeutic target in cancer with drug resistance.^34^

LNMAT2 is up‐regulated in lymph node‐positive bladder cancer tissues. Exosomal LNMAT2 derived from bladder cancer cells are transmitted to HLECs and promote lymphatic metastasis in bladder cancer. In vivo study, bladder cancer cells were injected to the footpads of nude mice and nude mice were treated with intratumoural injection with exosomes. Exosomes secreted by LNMAT2‐transfected cells promoted the lymph node metastasis of bladder cancer cells in nude mice. In vitro study, exosomes secreted by LNMAT2‐silenced cells inhibited HLECs tube formation and migration. It is indicated that decreased expression levels of exosomal LNMAT2 inhibit the malignant characteristics of bladder cancer cells; thus, LNMAT2 might be a therapeutic target for LN metastasis in bladder cancer.^35^

With the development on the investigation of promising lncRNA targets, the effective delivery of oligonucleotides targeting lncRNAs to the tumour cells is a key step in cancer treatment. There are several oligonucleotides delivery strategies, including lipid‐based carrier, polyethylenimine‐based carrier, protamine delivery, antibody‐mediated targeted delivery.^105^, ^106^, ^107^ Mice treated with pre‐miRNA mixed with liposomes had a higher expression of miRNA than the control ones.^108^ Protamine‐condensed siRNAs reduced the expression of cyclin D1 in vitro and in vivo.^109^ Effective delivery of oligonucleotides will accelerate the clinical application of the exosomal lncRNAs.

## 6 CONCLUSION

Exosomes are important factors involved in the progression and development of cancer. The cancer‐associated roles of exosomes mainly depend on their cargoes. The component proteins and RNAs exert different functions in pathological processes of cancer. Although lncRNAs participate in tumorigenesis through various mechanisms, the exosomal lncRNAs have same characteristics. Exosomal lncRNAs bind to miRNAs and release the genes targeted by miRNAs. Furthermore, they regulate gene expression through epigenetic modulation. Through various mechanisms of gene regulation, exosomal lncRNAs play critical roles in cancer cell proliferation, metastasis, drug resistance and angiogenesis. The significance of exosomal lncRNAs in cancer makes them eligible to serve as therapeutic targets for the treatment of cancers. As exosomes are widely existed in multiple fluids and those secreted by donor cells reflect the status of donor cells, the exosomal lncRNAs have a great value in prognosis and diagnosis of cancers.

Although there is a promising prospect in the clinical application of exosomal lncRNAs, some problems still remain unsolved. For instance, is there any difference between exosomal lncRNAs transferred to the recipient cells and those left behind in the recipient cells? As most exosomal lncRNAs exert similar functions in the recipient cells and in the donor cells, maybe these exosomal lncRNAs are labelled with some special modifications, which do not change the biological activity of lncRNAs. Secondly, what is the mechanism of exosomal fusion with recipient cells? There may be some molecules that regulate the interaction of exosomes and recipient cells. If we figure out the underlying mechanisms, we can easily manipulate the exosomes, pack wanted lncRNAs, proteins or DNA into the exosomes, and make exosomes to fuse with particular recipient cells. More detailed investigations are needed to interpret the complex process, which will enlarge the insight of biological functions of exosomes and facilitate the clinical application of exosomes.

## CONFLICTS OF INTEREST

We have no conflicts of interest to declare.

## AUTHOR CONTRIBUTIONS

WY conceived and drafted the manuscript. WY and ZM discussed the concepts of the manuscript. ZM drew the figures. LZ approved the version to be submitted.
